# Association of *Helicobacter pylori vacA* genotypes and peptic ulcer in Iranian population: a systematic review and meta-analysis

**DOI:** 10.1186/s12876-020-01406-9

**Published:** 2020-08-14

**Authors:** Masoud Keikha, Mohammad Ali-Hassanzadeh, Mohsen Karbalaei

**Affiliations:** 1 Antimicrobial Resistance Research Center, Mashhad University of Medical Sciences, Mashhad, Iran; 2 Department of Microbiology and Virology, Faculty of Medicine, Mashhad University of Medical Sciences, Mashhad, Iran; 3 Student Research Committee, Mashhad University of Medical Sciences, Mashhad, Iran; 4 Department of Immunology, School of Medicine, Jiroft University of Medical Sciences, Jiroft, Iran; 5 Department of Microbiology and Virology, School of Medicine, Jiroft University of Medical Sciences, Jiroft, Iran

**Keywords:** *Helicobacter pylori*, Iran, Peptic ulcer disease, *vacA* genotypes

## Abstract

**Background:**

*Helicobacter pylori* is accounted as the most etiologic agent for digestive disorders, in particular, the most important of them i.e. peptic ulcer and gastric cancer. In the recent years, association of *vacA* genotypes and gastrointestinal disorders has attracted a lot of attention. In present study, we assessed the correlation between *vacA* genotypes (s1, s2, m1, m2, s1m1, s1m2, s2m1 and s2m2) and development to peptic ulcer in Iranian population.

**Methods:**

In our study, first, 24 original articles containing of information of 3328 patients were evaluated. Statistical analysis was done by Comprehensive Meta-Analysis version 2.0 software (Biostat, Englewood, NJ, USA). In this regards, we used from fixed-effects model for analysis of data with low heterogeneity, while for analysis of data with high heterogeneity (*I*^*2*^ statistic index > 25%, Cochrane Q statistic *p* value < 0.05), random-effects model was used.

**Results:**

Abundance of each of s1, s2, m1, m2, s1m1, s1m2, s2m1, and s2m2 was estimated 36.24, 28.32, 42.90 29.86, 27.88, 32.34, 15.70, and 25.94%, respectively. According to the results, the m1, s1, and s1m2 genotypes were among the most prevalent genotypes among the Iranian patients, whereas, s2m1 genotype had the lowest frequency.

**Conclusions:**

Overall, 24 articles (total participants = 3328) were included in this comprehensive analysis. *H. pylori* infection rate were 90.26% in these cases, so that 33.65% of whom had peptic ulcer. Moreover, the abundance of each *vacA* genotypes including s1, s2, m1, m2, s1m1, s1m2, s2m1, and s2m2 was estimated as 36.24, 28.32, 42.90 29.86, 27.88, 32.34, 15.70, and 25.94% respectively. We demonstrated that there is a significant relationship between infection of stomach with m1, s1m1, and s2m1 genotypes and development to peptic ulcer disease.

## Background

In the gastrointestinal tract, peptic ulcer is induced following damage to mucosa and sub-mucosa tissues, which occurs due to the imbalance between invasive factors (secretion of gastric acid, pepsin, bile salts, increase of oxygen free radicals, consumption of non-steroidal anti-inflammatory drugs, and infection with *H. pylori*) and host defensive mechanisms (mucus, bicarbonate, prostaglandin, antioxidant, and blood circulation) [1–4]. While ulcers occur in gastric epithelium, is called gastric ulcer, and when lesions happen in the first part of duodenum, so called duodenal ulcer [5, 6]. The prevalence of peptic ulcer in different areas of world has been estimated 6–15%. Based on reports from The Ministry of Health and Medical Education (MOHME) of Iran, of all eight Iranians, one person has experienced peptic ulcer in his/her life, however, the frequency of duodenal ulcer is more than gastric ulcer [7–9]. According to review of the literature, infection with both *H. pylori* and non-steroidal anti-inflammatory drugs (NSAIDs) are considered as the most important causing agents for peptic ulcers, but the role of *H. pylori* is more prominent, so that this bacterium has isolated from 60 to 80% of peptic ulcer cases [9–11]. *H. pylori* and NSAIDs by independent mechanisms, but synergistically lead to severe inflammation and consequently peptic ulcer [12, 13]. *H. pylori* is a microaerophilic, S shaped, gram negative, and motile (by lophotrichous flagella) bacterium which is able to be colonized in human stomach [14]. Almost half of world population are infected to *H. pylori*, nevertheless, the rate of colonization in developing countries is more compared to western countries; most of population in developing countries first time infected with this bacterium in childhood ages [14, 15]. The International Agency for Research on Cancer (IARC) introduced this bacterium as the main causing enemy of gastric cancer [16, 17]. Also, this bacterium is accounted for some diseases such as primary gastric non-Hodgkin’s lymphoma, mucosa-associated lymphoid tissue lymphoma (MALT), gastritis, and peptic ulcer [18]. In recent years, virulence factors of *H. pylori*, and above all, cytotoxin-associated gene A (*CagA*) and vacuolating cytotoxin A (*VacA*) have more considered. *VacA* antigen is one of the well-known virulence factors of this pathogen which its gene, *vacA*, is present in all strains. The mosaic-like structure of *vacA* gene has both conserved and variable allelic sequences. These variable sequences are found in different regions from N-terminal side including signal sequence (s1 and s2) region, intermediate (i1 and i2) region, deletion (d1 and d2) region, and mid (m1 and m2) region, respectively. Whilst the cytotoxicity power of all genotypes differs from each other, in addition, two s1 and m1 regions in turn comprise several subtypes including s1a, s1b, s1c, m1a, m1b and m1c [14, 19–21]. This antigen through induction of cytoplasmic vacuolation and apoptosis in infected cells can lead to the death of host gastric epithelial cells [14, 22]. In addition, the toxin causes dysregulation of normal signaling pathway via happens such as alternation in the mitogen-activated protein kinases (MAPKs) pathway, polarization, suppression of proliferation and migration, as well as cytoskeletal changes [21, 22]. Evidence show that there is a significant relationship between the presence of *vacA* gene and progression of disease to peptic ulcer and gastric cancer [23, 24]. However, some studies have also rejected this correlation [25, 26]. Despite about 25 years from introduction of *VacA* antigen by Cover et al., but so far its properties has no recognized correctly. In Iran the abundance of peptic ulcer is about 41% (95% confidence interval, or 95% CI), which is much more compared to the global average (6–15%) [7, 19, 27]. Some characteristics such as high colonization by *H. pylori* (about 90%) and genetic diversity in *H. pylori* strains are influential in this phenomenon [28]. The main goal of this study was the determination of frequency of *vacA* (s1, s2, m1, m2, s1m1, s1m2, s2m1 and s2m2) alleles and also their relationship with creation of peptic ulcer in Iranian population.

## Methods

### Search strategy

**Fig. 1:**
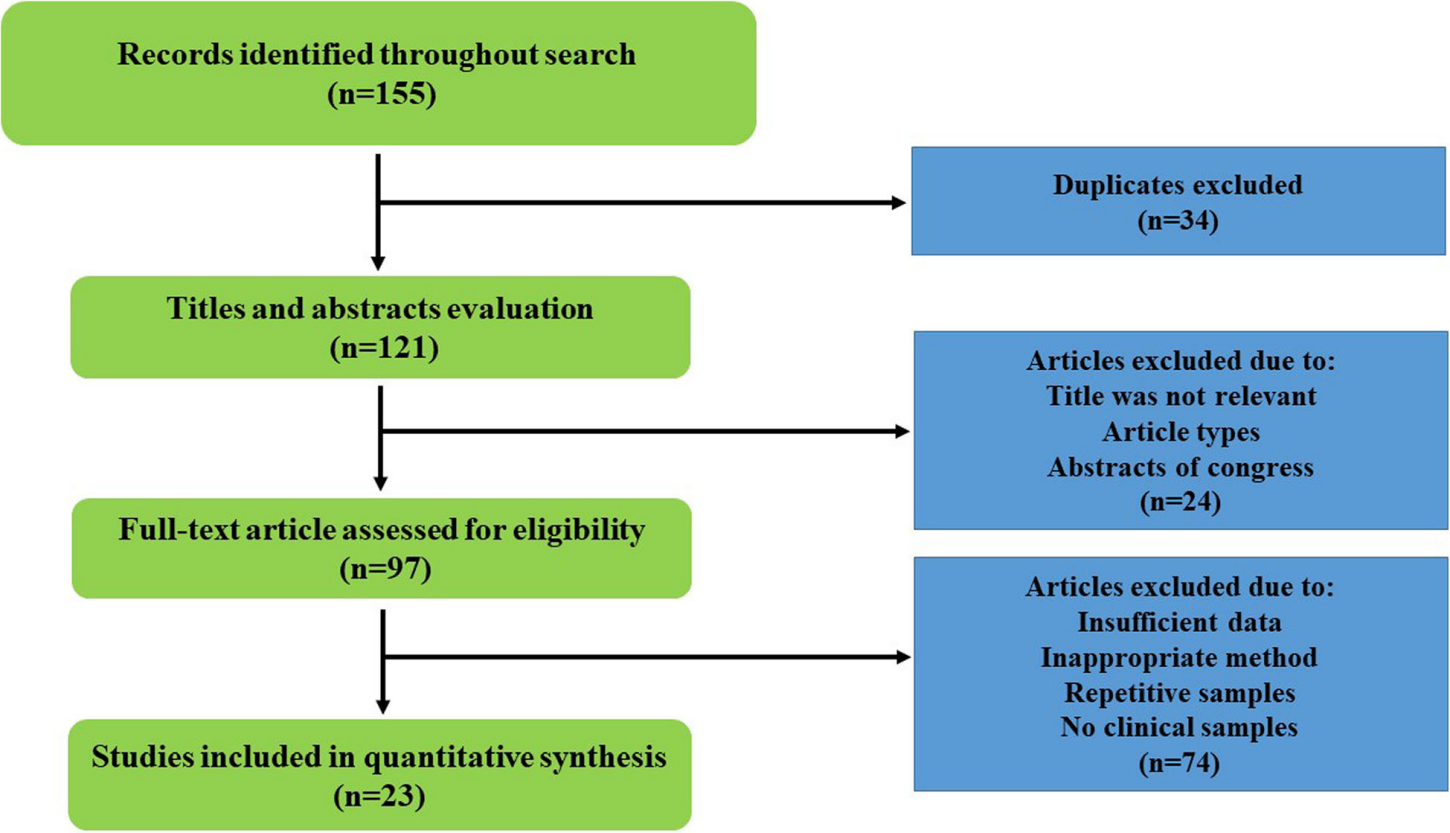
Flowchart of included and excluded articles

In the beginning, all studies (English and Persian) were received until March 2020 from global databases such as Google scholar, Scopus, PubMed, EMBASE, and also Iranian databases of IranMedex, SID, ISC. We used from keywords based on MeSH including “*Helicobacter pylori*”, “peptic ulcer”, “genotypes”, “Iran”, “*VacA* protein”, and “gastric ulcer”. In final, based on our inclusion criteria eligibility of articles was evaluated by two authors, separately (Fig. 1). The inclusion criteria were included original articles (cross-sectional, case–control, and cohort studies) associated with *vacA* genotypes (s1, s2, m1, m2, s1m1, s1m2, s2m1, and s2m2) in Iranian patients with peptic ulcer, and also original articles about the identification of *H. pylori* and its *vacA* genotypes. Whilst, other studies such as reviews, letter to editor, congress abstracts, laboratory animal’s studies, case reports, studies of other countries, ambiguous studies, and non-clinical studies were excluded from our research.

### Quality assessment and data extraction

Quality assessment of eligible studies was done based on the checklist. Afterwards, the most important information such as first author, publication year, city, age and gender distribution, number of *H. pylori* strains, number of peptic ulcer patients, and frequency of *vacA* genotypes (s1, s2, m1, m2, s1m1, s1m2, s2m1, and s2m2) was reported for each study (Table 1).

**Table 1:** Characteristics of included studies

First Author	Year	City	Peptic ulcer	*H. pylori* isolates	Age Female/Male	*cagA*	*VacA* genotypes								Ref
							s1	s2	m1	m2	s1m1	s1m2	s2m1	s2m2	
Dabiri	2017	Tehran	40	160	45.5 ± 1 81/79	26	24/109	16/51	12/48	28/112	4/30	20/79	8/18	8/33	[29]
Salari	2009	Tehran	50	50	45 21/29	NA	50/50	0/0	31/31	19/19	NA	NA	NA	NA	[30]
Salehi	2011	Tehran	54	100	9 53/70	NA	42/62	7/19	34/36	15/45	NA	NA	NA	NA	[31]
Doosti	2009	Shahrekord	150	178	NA NA	NA	NA	NA	NA	NA	24/38	56/96	0/7	3/39	[32]
Nahaei	2008	Tabriz	48	150	38.3 74/76	31	20/83	3/36	8/43	15/76	7/36	13/47	1/7	2/29	[33]
Douraghi	2010	Tehran	12	80	43.3 ± 1 56/60	NA	7/61	NA/19	3/26	9/54	8/26	12/35	0	8/19	[34]
Alikhani	2014	Hamadan	27	137	53 64/89	25	16/52	5/16	8/21	13/47	6/16	10/36	2/5	3/11	[35]
Sarvestani	2007	Shiraz	33	69	47.2 127/137	37	NA	NA	NA	NA	1/65	6/97	0/65	NA	[36]
Salehi	2009	Rasht	77	106	41 46/38	44	39/55	9/22	31/31	18/29	NA	NA	NA	NA	[37]
Abdollahi	2019	Kerman	6	120	38.2 98/93	4	6/45	NA	2/29	3/29	NA	NA	NA	NA	[38]
Havaei	2014	Isfahan	40	100	43 45/55	NA	40/100	NA	21/51	19/49	21/51	19/49	NA	NA	[39]
Ghotaslou	2013	Tabriz	62	115	NA NA	47	48/82	47/79	14/21	48/94	13/19	35/63	1/2	47/79	[40]
Khodaii	2010	Tehran	73	141	41.4 ± 6 99/58	56	57/97	16/43	32/47	41/93	16/29	37/64	6/14	14/33	[41]
Dabiri	2009	Tehran	13	124	44.3 65/59	6	8/50	5/24	6/22	7/52	2/15	6/35	4/7	1/17	[42]
Khodaii	2013	Tehran	83	157	41.1 58/99	56	57/97	16/43	32/47	41/93	16/29	37/64	6/14	14/33	[43]
Rezaeian	2012	Jahrom	38	164	47 58/79	34	NA	NA	NA	NA	18/63	12/73	1/6	7/22	[44]
Sedaghat	2014	Kashan	8	37	44.6 ± 1 123/99	4	4/20	3/13	1/9	6/23	0/6	4/15	1/2	2/8	[45]
Rafeey	2013	Tabriz	4	33	8.28 NA	2	1/37	1/20	0/16	2/41	NA	NA	NA	NA	[46]
Souod	2013	Jahrom	38	201	47 ± 1 85/79	34	15/135	8/29	19/67	19/97	23/108	10/73	1/6	7/22	[47]
Pajavand	2015	Kermanshah	20	96	46 41/55	NA	19/47	1/49	3/10	17/86	NA	16/38	NA	1/47	[48]
Jafari	2008	Tehran	19	96	48 ± 1 29/26	15	10/66	8/27	4/30	13/59	2/22	7/40	2/8	6/19	[49]
Mohammadi	2003	Tehran	29	132	37.6 65/67	NA	23/93	4/36	8/42	17/74	6/35	14/43	0/4	3/31	[50]
Falsafi	2015	Tehran	34	172	9.5 ± 2 72/37	NA	NA	NA	NA	NA	8/34	14/59	5/26	7/40	[51]
Sarvestani	2006	Shiraz	61	286	45.3 ± 1136/150	54	NA	NA	NA	NA	21/81	33/110	NA	11/73	[52]

### Data analysis

In the present meta-analysis, we estimated abundance of each *vacA* genotypes in Iranian patients with peptic ulcer. Possible relationship between each *vacA* genotypes and development of peptic ulcer was measured by Odds Ratio (OR) with 95% CIs [18]. Statistical analysis was done by Comprehensive Meta-Analysis version 2.0 software (Biostat, Englewood, NJ, USA). In this regards, we used from fixed-effects model for analysis of data with low heterogeneity, while for analysis of data with high heterogeneity (*I*^*2*^ statistic index > 25%, Cochrane *Q* statistic *p* value < 0.05), random-effects model was used. On the other hand, for estimation of publication bias, the Egger’s regression model was employed.

## Results

Following initial searches, 155 articles was received from various databases. Finally, after study of titles, abstracts, and conformity with eligible criteria, 24 articles met inclusion criteria and were analyzed in present study [29–52]. Studies were done during 2003–2019, and from Tehran (43.4%), Tabriz (13%), Shiraz and Jahrom (8.6%), and Shahrekord, Kerman, Kermanshah, Rasht, Isfahan, and Hamadan (each, one study) cities (Table 1).

**Fig. 2:**
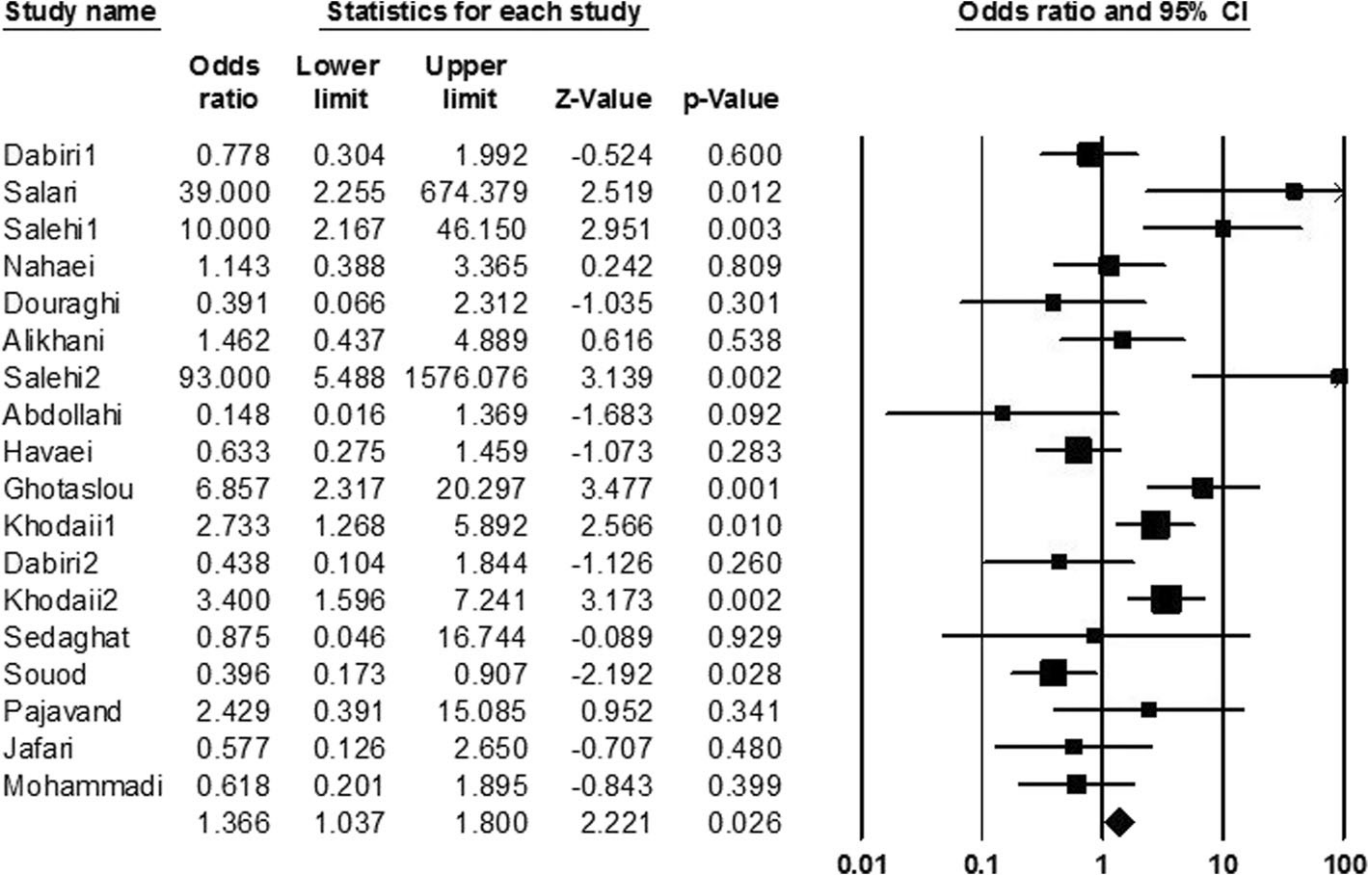
Forrest plot of the *vacA* genotype m1.The association between *vacA* genotype m1 and development to peptic ulcer in Iranian populations

**Fig. 3:**
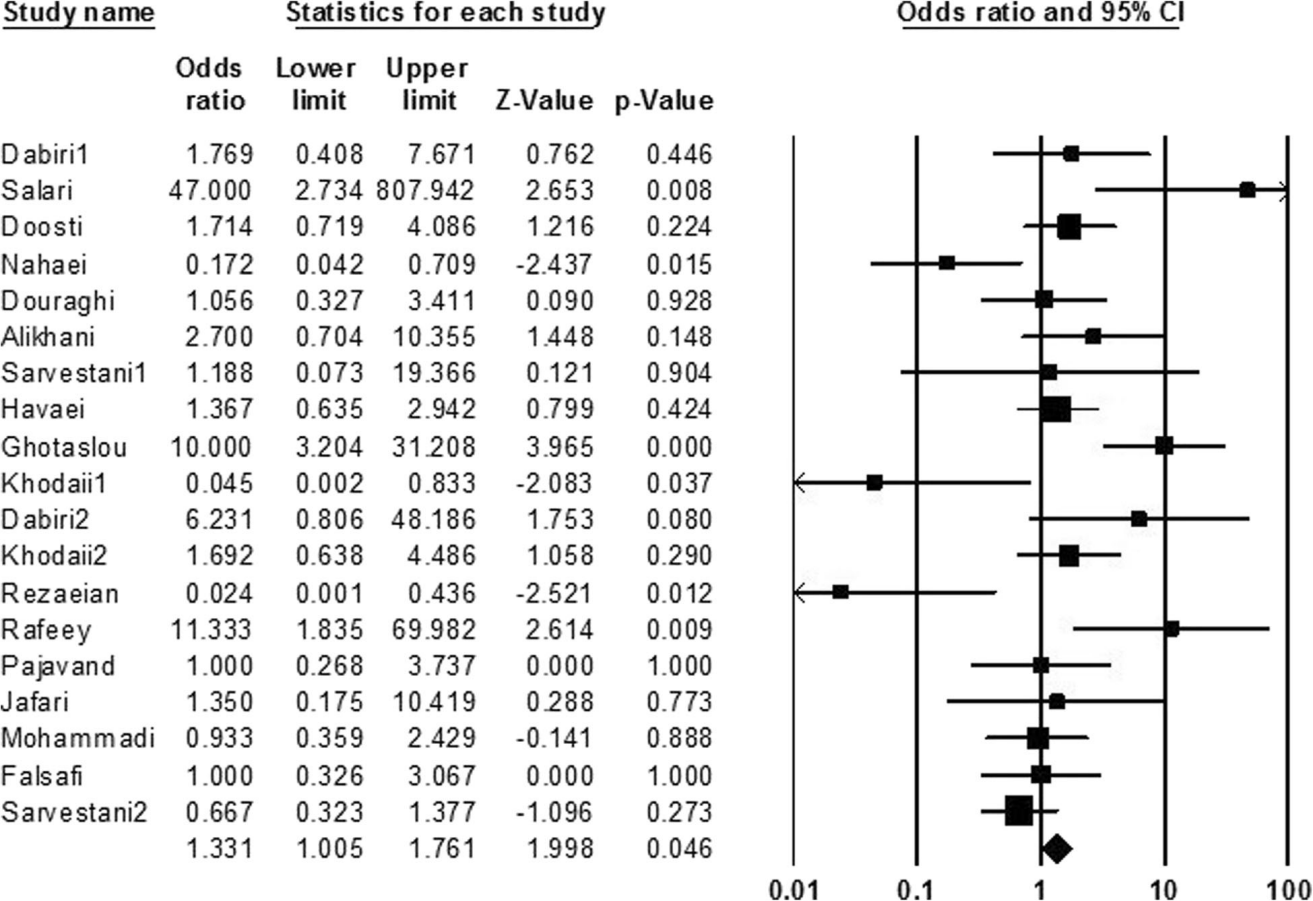
Forrest plot of the *vacA* genotype s1m1.The association between *vacA* genotype s1m1 and development to peptic ulcer in Iranian populations

**Fig. 4:**
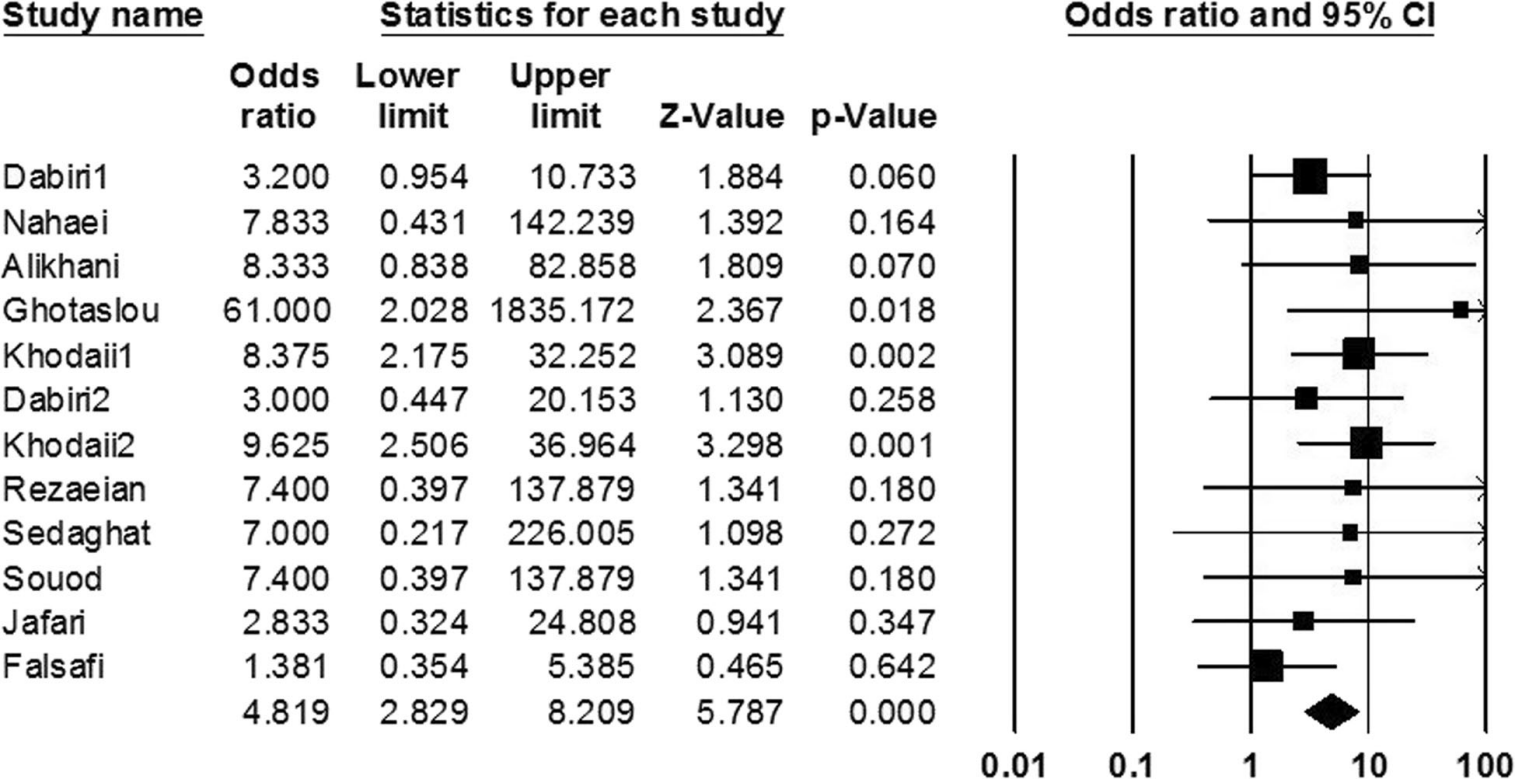
Forrest plot of the *vacA* genotype s2m1. The association between *vacA* genotype s2m1 and development to peptic ulcer in Iranian populations

In the present meta-analysis, information of 3328 patients was evaluated which of them, about 55.05% were men, and about 44.95% of them were women; average age of studied population was about 41.1 ± 2. Among all cultured samples, *H. pylori* was isolated from 3004 (90.26%) cultivated biopsies, and also 1120 (33.65%) cases had peptic ulcer. The result of cultured samples of other patients (324 cases) with peptic ulcer was negative. Peptic ulcer in patients with negative culture could be due to administration of nonsteroidal anti-inflammatory drugs (NSAIDs), and or non-growth of this fastidious bacterium on the culture media. In addition, among of patients with peptic ulcers, frequency of duodenal ulcer cases was more than gastric ulcer ones. Abundance of each of s1, s2, m1, m2, s1m1, s1m2, s2m1, and s2m2 was estimated 36.24, 28.32, 42.90 29.86, 27.88, 32.34, 15.70, and 25.94%, respectively. Regarding this, it was demonstrated that *vacA* genotypes such as m1, s1, and s1m2 were the most prevalent *vacA* alleles among the Iranian patients with peptic ulcer. Finally, based on statistical analysis estimations, a significant relationship was observed between infections by m1, s1m1, and s2m1 alleles and development to peptic ulcer (OR 1.36, 1.24 and 4.82 respectively) in Iranian patients (Figs. 2, 3, and 4).

**Fig. 5:**
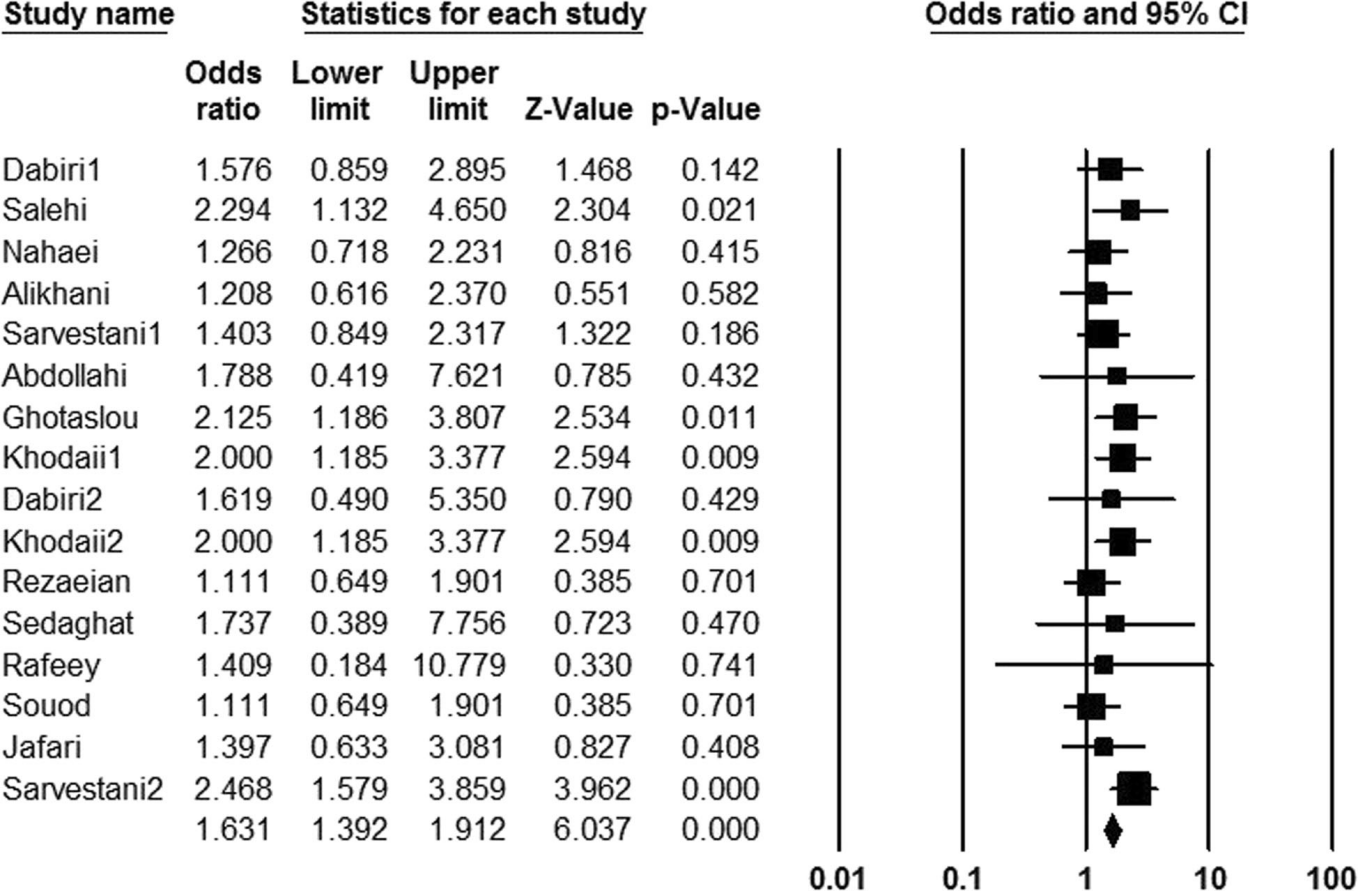
Forrest plot of the association of coexistence vacA/cagA with development of disease to peptic ulcer in Iranian populations

The pooled ORs and 95% CIs for relationship between each of *vacA* genotypes and susceptibility to peptic ulcer in the Iranian population is summarized in Table 2. Furthermore, the frequency of coexistence of *vacA* and *cagA* genotypes in patients with peptic ulcer was evaluated about 33.35%. We found a meaningful relationship between infection with *vacA* and *cagA* positive strains of *H. pylori* and development to peptic ulcer (OR: 1.63, 1.39–1.91; *Q-value*: 12.15; *I*^*2*^: 0.00; *p* value: 0.00 and Egger’s regression: 0.53) (Fig. 5). In addition, frequency of *cagA* gene in s1, s2, m1, m2, s1m1, s1m2, s2m1 and s2m2 genotypes was estimated 46.08, 11.14, 12.34, 35.24, 20.18, 50, 6.62, and 15.96%, respectively. Thus, s1m2, s1, and m2 genotypes were the most prevalent genotypes which harboring *cagA* gene, respectively. However, due to limited information, we could not evaluate the frequency of *cagA* gene in each of *vacA* genotypes isolated from Iranian patients with peptic ulcer.

**Table 2:** Summary of OR with 95% CIs for comparison of all *vacA* genotypes with each other

*vacA* genotypes	Odds Ratio		Heterogeneity		Egger’s regression
	95% CIs	*p* value	*Q*-value	*I*^*2*^-squared	
s1	0.35; 0.28–0.44	0.00	69.13	75.40	0.12
s2	1.21; 0.86–1.62	0.20	30.42	53.98	0.23
m1	1.36; 1.03–1.80	0.026	62.68	72.88	0.02
m2	0.42; 0.34–0.53	0.00	97.55	81.54	0.05
s1m1	1.33; 1.00–1.76	0.046	52.54	65.74	0.46
s1m2	0.73; 0.60–0.90	0.003	58.24	69.09	0.02
s2m1	4.81; 2.82–8.20	0.00	8.48	0.00	0.53
s2m2	1.28; 0.94–1.72	0.10	25.40	37.02	0.03

## Discussion

In the present study, we estimated the frequency of peptic ulcer about 33% in Iranian patients infected with *H. pylori*, which despite of higher prevalence than global average, but confirms previous studies from Iran. Perhaps this phenomenon is to be due to some factors such as genetic properties of Iranian population, life style, and characteristics of circulating strains in Iran [7, 53]. Among the patient possessed peptic ulcer, frequency of duodenal ulcer was more than gastric ulcer, and also, the majority of patients were male. Further, like previous studies, age average of studied cases was measured about 41 years old [12, 26]. Regarding the present results, *vacA* genotypes m1, s1, and s1m2 were the three most prevalent isolated genotypes from Iranian patients involved with peptic ulcer. As well as, we demonstrated that there is a significant relationship between infection by strains containing m1, s1m1, and s2m1 genotypes and progression to peptic ulcer. Besides, in this meta-analysis, frequency of strains containing coexistence of *vacA* and *cagA* genes in peptic ulcer patients was assessed about 33.35%. We showed that there is a meaningful relationship between infections by *cagA*/*vacA* positive strains and development to peptic ulcer. *H. pylori* possesses some unique characteristics which cause to persist of bacterial infection in acidic condition of stomach and also evading from immune system [53, 54]. The colonization by this bacterium is different in various regions worldwide; for example, in Iran, 90% of population are infected with *H. pylori* [55]. Nevertheless, most of infected people remain as an asymptomatic carrier throughout the life of themselves; peptic ulcer and gastric cancer happen in 10–15 and 2% of infected cases, respectively [53, 55]. It seems that the infection by *H. pylori* can be related to socioeconomic status which in turn effects on papulation’s lifestyle. The outcomes of clinical manifestations of this bacterium are influenced by three factors, severity of strains, host genetics, and environment [56]. Reciprocally and over the time the stomach status such as acidity, buffering and mucus content can be affected by lifestyle factors such as diet, food habits, alcoholism, oral hygiene, water hygiene, personal hygiene, and so on [57]. In this regard, low socioeconomic status, overcrowding, poor hygiene, as well as living in the developing countries are considered as risk factors for progressing of primary infection to more severe complications including chronic gastritis, peptic ulcer, and also gastric cancer [58]. Overall, the prevalence of infections by this bacterium in developing countries, in particular in the countries with low socioeconomic status and poor health management (> 80%) is much higher than the developed ones (< 40%), [59]. Based on Safak et al., the prevalence of *cagA*- and *vacA*s1-positive strains in patients with active chronic gastritis was more than non-active chronic gastritis (45.8% vs 21.6 and 78.0% vs 40.5%, respectively) [60]. On the other hand, it seems that host genetic properties and pathogenicity power of *H. pylori* strains are as two determining factors in the onset of disease and final outcomes [53, 61]. According to review of the literature, global prevalence of peptic ulcer has been estimated about 10%, and this bacterium isolated from 90 to 100% and 60–90% cases of duodenal ulcer and gastric ulcer, respectively [53]. Nonetheless, frequency of peptic ulcer in Iran is much more than world average, which is related to host genetic characteristics and virulence factors of bacterium [7, 53]. Both surface antigens and cytotoxic enzymes such as *VacA* and *CagA* are accounted as the two main virulence factors of *H. pylori* [14, 24]. Based on previous meta-analysis, some virulence factors of *H. pylori* e.g. *OipA*, *BabA*, *DupA*, *IceA*, *CagA*, and *VacA* are related to progress to peptic ulcer disease [24, 53, 62–68]. Also, it seems that type of colonization can be effective in formation of peptic ulcer; in general, duodenal ulcer is create following antral colonization, but gastric ulcer is the result of corporal and pan-gastritis [53, 61, 68, 69]. Although *vacA* gene is present in all *H. pylori* strains, but its functional protein, *VacA* toxin, expressed in only 50% of those. The *VacA* protein forms a channel in membrane of bacterium, which be able to uptake of different ions and metabolites to the inside the cytoplasm, and causes to survival of bacterium in stomach mucosal layer. Endocytosis of *VacA* into the host cell leads to some events such as vacuoles formation, releasing cytochrome c from mitochondria, and apoptosis. In addition, *VacA* toxin by impressing on different receptors leads to alteration in signaling pathways of MAPK/p38 and extracellular signal-regulated kinases 1 and 2 (ERK1/2) [19, 69–72]. Functional weight of *VacA* toxin is about 88 kDa, and forms two subunits p33 and p55. The p33 domain which contains residues 1–33 in N-terminal region (as signal sequence) of *VacA* toxin, and creates vacuole in host cell [70, 73]. On the other, p55 domain acts as binding domain of toxin to the cell surface [70]. The length of *vacA* gene is 3860–3940 bp, and contains both conserved and variable regions. Nowadays, it has been cleared that the variable regions can be effective in variations of *vacA* gene expression, and directly are related to clinical outcomes of infection by *H. pylori* [70, 73]. For example, McClain et al. in 2001 showed that the hydrophobic amino acids near the cleavage site of s2, could integrated the *VacA* toxin with host cell membrane [74]. According to literature, *vacA* gene possesses variable sequences in s (s1 and s2) and m (m1 and m2) regions. It is notable that *vacA* s1m1 has the most expression rate, and therefore high vacuolating, but *vacA* s1m2 is a moderate vacuolating genotype, as well as *vacA* s2m2 is not toxic, and finally, *vacA* s2m1 genotype is rare and non-toxic [75–78]. Recently, two additional variable regions, i1/i2 and d1/d2, have recognized in m region, and also, each of s1 and m1 regions subdivided to different types such s1a, s1b, s1c, m1a, m1b and m1c [79, 80]. In the recent present, we showed that there is a significant relationship between infection by *cagA* positive *H. pylori* strains and peptic ulcer disease. Given that studies in this field, expression of *cagA* gene leads to increase of pathogenicity, and directly related to severity of diseases of bacterium [53]. Our study confirmed previous studies [65, 81]. Moreover, we demonstrated that m1, s1m1, and s2m1 genotypes have direct correlation with peptic ulcer in Iranian population. But, due to limit information about the both d and i genotypes, we could not assess the effect of these genotypes on development of infection to peptic ulcer. In 2014, Basiri et al. showed that the infection by d1 genotype of *vacA* gene raises the risk of primary infection towards gastric adenocarcinoma and peptic ulcer in Northwestern of Iran [82]. In another study in 2014, Mottaghi et al. studied on correlation between infection by i1 allele and development of infection into the gastric cancer and peptic ulcer in Azerbaijan, Iran; they found that *vacA* i1 genotype is significantly related to gastric cancer, however in their study, they did not find a meaningful relationship between infection by *vacA* i1/2 alleles and peptic ulcer gastric cancer diseases [83]. According to various European studies, it has been demonstrated that there is a significant correlation between *vacA* genotypes of s1 and m1 with *H. pylori*-related gastrointestinal diseases [75, 80, 84–87]. It is notable that due to decrease or absence of vacuolating activity, s2 and m2 genotypes rarely are related to peptic ulcer [80]. In our analysis, we observed a similar correlation about frequency of s1 and m1 alleles in patients involved by peptic ulcer with other studies, which is due to some properties of these bacterial strains such as increased binding capacity, vacuolating activity, and alternation in normal signaling pathway [75]. In addition, it is known that the origin of Iranian circulating strains is like to Western countries, in that, in 2010 Latifi-Navid et al. proved that the origin of Iranian strains is belonging to European *H. pylori* (*hpEurope*) strains. It seems that following migration of European to Iran, the *hpEurope* strains have been transferred to Iran, and this phenomenon can be effective in similarity of results of both our studies and Western countries [88]. Overall, most recent studies have confirmed an intimate relationship between infection by s1m1 strains and progression to gastrointestinal diseases [24, 80, 89, 90]. In 2005, Martins et al. represented that a significant relevance between colonization by *vacA* genotype s1m1 and peptic ulcer in Brazilian population [79]. Likewise, several separate studies have confirmed relationship between *H. pylori vacA* s1m1 infection and peptic ulcer [26, 79, 89, 90]. Based on our results, s1, m1, and s1m2 were proposed as the most prevalent genotypes in peptic ulcer disease. In a study that was done by Sugimoto et al., in 2009, they demonstrated that the frequency of s1 and m1 genotypes in Middle-East patients is more than 50%; they found that s1m1 and s2m1 were the most and lowest common genotypes in Middle-East regions, respectively, which in turn their results were according to our results [91]. While, based on Sugimoto et al. study, s1, m1, and s1m1 genotypes were related to peptic ulcer, but in our findings, m1, s1m1, and s2m1 were accounted as risk factor for peptic ulcer. It may be due to difference in distribution of patients; we only studies on Iranian patients’ samples [91]. We declared that there is a direct association between *vacA* s2m1 with peptic ulcer in Iranian patients; this is while, it seems that the strains which harboring s2m1 are non-toxic, or low capacity of vacuolation, and this finding was challenging [92]. Although due to limit information, we could not evaluate the presence of other virulence factors in s2m1 strains, it seems that these strains possess *cagA* gene or other required virulence factors for development to peptic ulcer. However, *H. pylori* strains from patients involved to peptic ulcer and gastric cancer in some regions such as Mexico, Latin America, Africa, and Western countries were harboring *vacA* s2m1 genotype [86, 87, 93]. In the same year, Sugimoto et al. demonstrated that abundance of *vacA* s2m1 in Mexican population is about 12.2% [80]. Furthermore, Zhang et al. displayed that infection by *vacA* s2m1 genotype and duodenal ulcer are significantly related with each other (OR: 2.30; 95% CIs: 1.17–4.50) [89]. Yet, it needed to more study about the effect of *vacA* s2m1 strains on creation of peptic ulcer. In summary, based on our and other meta-analysis results, the most common *vacA* genotypes involved in progressing to peptic ulcer disease which are listed in Table 3 [23, 66, 91].

**Table 3:** The most common *vacA* genotypes involved in progressing to peptic ulcer

Most common genotypes associated with peptic ulcer disease	Iran	Europe	Southeast Asia	Middle East
s1	+	+	+	+
s2	–	–	–	–
m1	+	+	+	+
m2	–	–	–	–
s1m1	–	–	–	–
s1m2	+	–	–	–
s2m1	–	–	–	–
s2m2	–	–	–	–
i1	–	–	+	–

## Conclusions

In general, in the present study we showed that there is a significant relationship between *vacA* genotypes m1, s1m1, and s2m1, and development of infections caused by *H. pylori* to peptic ulcer disease in Iranian population. In addition, based on collected information from Iran and other three regions including Europe, Southeast Asia, and Middle East, it seems that two main genotypes s1, and m1 are the most prevalent genotypes for progressing of primary infection to peptic ulcer. Finally, the limitations of our study were including: 1) limited information of patients; 2) spatial constraints, so that most of the studies were conducted in Tehran; 3) limited information of *vacA* d and i alleles; 4) limited information of *cagA* and other bacterial virulence factors; 5) publication bias in some studies.

## Data Availability

All data generated or analysed during this study are included in this published article and its supplementary information files.

## Abbreviations

*H. pylori*: *Helicobacter pylori*
MOHME: Ministry of Health and Medical Education
NSAIDs: Non-steroidal anti-inflammatory drugs
IARC: International Agency for Research on Cancer
MALT: Mucosa-associated lymphoid tissue lymphoma
CagA: Cytotoxin-associated gene A
VacA: Vacuolating cytotoxin A
MAPKs: Mitogen-activated protein kinases
